# Volatile Organic Compound Metabolites Are Found in the Urine of Breastfed Infants Whose Mothers Use Cannabis: The Lactation and Cannabis (LAC) Study

**DOI:** 10.3390/ijerph23070905

**Published:** 2026-07-15

**Authors:** Shelby Samartino, Benjamin Blount, Christopher Reese, Lanqing Wang, David R. Gang, Celestina Barbosa-Leiker, Elizabeth A. Holdsworth, Janet E. Williams, Mark A. McGuire, Michelle K. McGuire, Courtney L. Meehan

**Affiliations:** 1 Department of Anthropology, Washington State University, Pullman, WA 99164, USA; shelby.samartino@wsu.edu; 2 Division of Laboratory Sciences, National Center for Environmental Health, Centers for Disease Control and Prevention, Atlanta, GA 30341, USA; bkb3@cdc.gov (B.B.); wri5@cdc.gov (C.R.); lfw3@cdc.gov (L.W.); 3 Institute of Biological Chemistry, Washington State University, Pullman, WA 99164, USA; gangd@wsu.edu; 4 College of Health and Human Development, California State University, Fullerton, CA 92831, USA; cbarbosa-leiker@fullerton.edu; 5 Department of Anthropology, The Ohio State University, Columbus, OH 43210, USA; holdsworth.35@osu.edu; 6 Department of Animal, Veterinary and Food Sciences, University of Idaho, Moscow, ID 83844, USA; janetw@uidaho.edu (J.E.W.); mmcguire@uidaho.edu (M.A.M.); 7 Margaret Ritchie School of Family and Consumer Sciences, University of Idaho, Moscow, ID 83844, USA; smcguire@uidaho.edu

**Keywords:** lactation, breastfeeding, cannabis, volatile organic compounds, infant exposure

## Abstract

**Highlights:**

**Public health relevance—How does this work relate to a public health issue?**

- This study examines the relationship between maternal cannabis use during lactation and the risk of exposure to potentially harmful volatile organic compounds (VOCs) among breastfeeding infants.

**Public health significance—Why is this work of significance to public health?**

- This work addresses a gap in the perinatal literature on associations between cannabis use while breastfeeding and VOC exposure in infants.
- Results can be used in perinatal healthcare practices and to assist mothers in making informed decisions about cannabis use during lactation.

**Public health implications—What are the key implications or messages for practitioners, policy makers and/or researchers in public health?**

- Smoking-related VOC metabolites are found in the urine of infants whose mothers use cannabis during breastfeeding.
- Infants of mothers who smoke cannabis versus vape cannabis have higher concentrations of smoking-related VOC metabolites in their urine.

**Abstract:**

Cannabis use among breastfeeding women is increasing. While studies have shown that cannabinoids transfer into human milk, whether breastfed infants of mothers who use cannabis are exposed to volatile organic compounds (VOCs) is not known. Here, we characterized VOC metabolite concentrations in urine produced by exclusively breastfed infants whose mothers used cannabis. Relationships between infant urinary VOC metabolite concentrations and milk delta-9-tetrahydrocannabinol (∆^9^-THC) concentrations and cannabis use modality (smoking/vaping) were documented. Twenty mother–infant dyads (<6 months postpartum) living in Washington and Oregon were enrolled in the Lactation and Cannabis (LAC) Study, and nineteen were included in the current study. Mothers collected baseline infant urine and milk samples (following ≥12 h of cannabis abstention) and three infant urine and five milk samples over 8–12 h following cannabis use. Sixty-five urine samples were analyzed for VOC metabolite concentrations, and 111 milk samples were previously analyzed for ∆^9^-THC. Results indicate detectable levels of 24 VOC metabolites in infant urine. Non-baseline infant urine had greater concentrations of 15 VOC metabolites than matched baseline samples (*p* < 0.05). Urinary 2CyEMA (acrylonitrile metabolite) and 2CaEMA (acrylamide metabolite) concentrations were positively correlated with ∆^9^-THC concentrations in milk. Urinary 2CyEMA concentrations were higher in infants whose mothers primarily smoked (including those who smoked and vaped) compared to those who exclusively vaped (B = −0.637, SE = 0.317, *p* = 0.044). 2CaEMA concentrations were also greater in the urine produced by infants whose mothers reported any cannabis smoking versus only vaping on the study day (B = −4.06, SE = 1.42, *p* = 0.004). Results indicate that infants whose mothers use cannabis have measurable VOC exposure. Further research is needed to assess the potential health implications, if any.

## 1. Introduction

Cannabis use among pregnant and breastfeeding women is increasing [1]. Cannabis is the most used illicit (U.S. federally defined) drug during pregnancy, with self-reported estimates ranging from 2 to 5% among pregnant women, but as high as 15–30% in low-income and urban populations [2,3,4]. Additionally, there is a growing perception among breastfeeding women that cannabis use during lactation is not risky [5]. Yet there is a dearth of data on potential effects, if any, of milk-borne cannabinoids on human milk composition and the developing infant [6,7,8]. Furthermore, it is still unclear whether and/or how byproducts of maternal cannabis use may impact the infant.

Nonetheless, the growing literature indicates that cannabinoids transfer into human milk [9,10,11], with one study reporting detectable levels of delta-9-tetrahydrocannabinol (∆^9^-THC) in milk for up to 48 h after use [6] and as long as up to >6 weeks [12]. One study has examined associations between cannabis use and concentration of other milk components such as macronutrients [13], and two studies have assessed the impact of maternal cannabis use during breastfeeding on infant development [14,15]. Regardless, the transfer of cannabinoids to the infant via human milk is not the only potential concern. Breastfed infants may also be exposed to cannabis’ secondary smoke compounds (volatile organic compounds; VOCs) either directly via human milk and/or through environmental exposure through secondhand (smoke streams from other people) and thirdhand (deposits on surfaces) smoke. To our knowledge, no studies have yet examined VOC exposure to infants when their mothers use cannabis. Despite the steady increase in cannabis use [1], there remains limited research, albeit some [16], on the potential exposure to these compounds via cannabis inhalation.

This is important because long-term VOC exposure increases the risk of cancer and birth defects [17,18,19]. As with tobacco users, smoking is the primary modality of cannabis use for habitual users [20]. There is growing consensus that cannabis smoke, like tobacco smoke, contains harmful chemical compounds, including carcinogenic VOCs [21,22,23,24]. For instance, N-acetyl-S-(2-cyanoethyl)-L-cysteine (2CyEMA), a urinary metabolite of acrylonitrile—a known carcinogen—was elevated in the urine of adults who used cannabis in the previous 30 days compared to adults who had not used cannabis [16]. In urine collected before and after smoking, vaping, or eating cannabis, smoking a cannabis joint markedly increased 2CyEMA concentrations within 10 min in occasional users, but vaping or eating cannabis did not alter 2CyEMA levels. 2-carbamoylethyl mercapturic acid (2CaEMA, N-Acetyl-*S*-carbamoylethyl-L-cysteine) is a urinary metabolite biomarker for exposure to acrylamide, a compound that can be found in certain foods and is also a carcinogenic VOC found in cigarette smoke [25]. Because acrylamide is commonly found in cigarette smoke, it may also be found in cannabis smoke, making further investigation of these smoking-related VOCs in cannabis users vital.

Here, we (1) characterized urinary VOC metabolite concentrations in breastfed infants of mothers who use cannabis; (2) investigated associations between VOC metabolite concentrations of urine produced by breastfed infants and cannabinoid concentrations in the milk produced by their mothers; and (3) assessed associations between infant urinary VOC metabolite concentrations and maternal frequency and mode of cannabis use (any smoking vs. only vaping) and frequency-of-use patterns. Our hypotheses were as follows.

(a) Infants breastfed by mothers who use cannabis have detectable concentrations of VOC metabolites in their urine.
(b) VOC metabolite concentrations in urine produced by infants breastfed by women who use cannabis and ∆^9^-THC concentrations of the milk they produce are positively associated.
(c) Infants of mothers who smoke cannabis have greater VOC metabolite concentrations in their urine than infants of mothers who exclusively vape.
(d) Frequency of maternal cannabis use is positively associated with VOC metabolite concentrations in their breastfed infants’ urine.

## 2. Materials and Methods

### 2.1. Parent Study

This study builds upon data collected as part of the Lactation and Cannabis (LAC) Study that examined cannabis use during lactation and the pharmacokinetics of cannabinoids in the mother–infant dyad [5,10]. The study, which was a case–control repeated measures observational study, enrolled 20 breastfeeding women who frequently used cannabis (≥1× weekly) and their infants. To participate, mothers and their infants needed to self-report as healthy (e.g., no fever, cough, infection in the past 7 days) and reside in Washington or Oregon. Mothers had to be >21 years of age, have delivered a full-term infant within the past 6 months, be breastfeeding or pumping >5 times daily, and not using illicit drugs (e.g., opioids). The parent study was reviewed and approved by Washington State University Institutional Review Board (protocol #19087). A Certificate of Confidentiality was obtained from the National Institutes of Health. The analysis presented here on deidentified samples and data were determined by Washington State University to not meet the definition of human subjects research (IRB# 20318) and was conducted in accordance with applicable federal law and Centers for Disease Control and Prevention (CDC) policy.

### 2.2. Metadata

A RedCap^TM^ [26] survey was distributed to participants to collect demographic (e.g., age and time postpartum), substance use history, and cannabis use data (Daily Sessions, Frequency, Age of Onset, and Quantity of Cannabis Use Inventory [DFAQ-CU] [27]). The questions on cannabis use included participants’ categorical estimation of their current cannabis use frequency (12 possible options ranging from less than once a year to more than once a day) and primary method of cannabis use (options included joints, blunts, hand pipe, bong, hookah, vaporizer, edibles, or other) and their primary form of cannabis used (marijuana, concentrates, edibles, or other). We defined “smoking” to include any mothers who reported smoking on the study, including those who smoked and vaped.

### 2.3. Milk and Urine Collection Procedures and Timing

**Figure 1:**
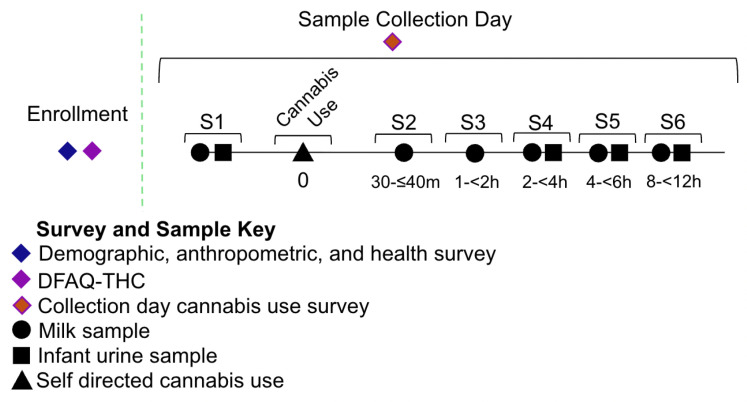
Sampling and data collection protocol and timeline. Sample 1 (S1) was collected at baseline before cannabis use; S2–S6 were collected at timed intervals after cannabis use. Participants recorded cannabis use throughout the sample collection day via a survey.

As previously described [10] mothers collected 6 milk samples across a study day (Figure 1). Sample one (S1) was a baseline milk sample collected after ≥12 h of cannabis abstention. After baseline sample collection, at a time of their choosing, mothers used cannabis. Cannabis was not provided, and participants were instructed to use cannabis how they normally would use it. Participants used their own products and recorded product information and use information. Following cannabis use, mothers collected an additional 5 milk samples at 30–40 m, 1- < 2 h, 2- < 4 h, 4- < 6 h, and 8–12 h after initial cannabis use. After sanitizing the breast with a cleaning wipe, mothers pumped a full breast expression at each sample collection using their own pumps and bottles, following a detailed cleaning of the components each time, as per provided instructions. Milk was collected into collection bottles, swirled, and aliquoted [10]. Figure 1 provides an overview of the sample and data collection protocol.

Participants were instructed to collect urine samples at baseline (after ≥12 h of maternal cannabis abstention) and approximately 2, 4, and 8–12 h after the initial maternal cannabis use. If the infant had not urinated at the predetermined collection time, the sample was collected as soon as possible thereafter, and participants recorded the collection time. To collect infant urine, mothers followed instructions that directed them to clean their infant’s bottom, place a new (provided) diaper on the infant, place sterile absorbent cotton balls (Curad, Northfield, IL, USA) into the diaper, and check the diaper frequently to avoid fecal contamination. A strip of waterproof hypoallergenic tape [Kendall™/Covidien Waterproof Tape (Cardinal Health, Dublin, OH, USA)] was placed in the provided diaper to ensure the cotton balls would absorb the urine before the diaper. Following urination, cotton balls were placed into a provided syringe (VRW International, Tuttlingen, Germany), and the urine was subsequently dispensed into sterile polypropylene cryotubes (VWR, BioCision LLC, San Rafael, CA, USA). Mothers placed samples in their home freezer and called study personnel at the end of the study day for sample retrieval within 24 h. Frozen samples were transported on ice and stored at −80 °C until VOC analysis by the Division of Laboratory Sciences at the CDC.

### 2.4. Milk Cannabinoid LC-MS Analysis

Analysis of cannabinoids was previously performed and described [10], which is closely based on the method reported by Britch et al. [28] as well as an application note by Young and Martin [29]. Holdworth et al. [10] contains, in addition to the original cannabinoid concentration data utilized again for this study, a detailed protocol in its Supplementary Information that provides the steps involved in the cannabinoid analysis method utilized for that and this study, including instrument parameters, grades of solvents used, source of cannabinoid standard compounds, including deuterated counterparts and their use as appropriate internal standards to anchor quantitation, and how standard calibration curves were set up to enable sensitive detection and quantification of cannabinoids, including Δ^9^-THC.

### 2.5. Infant Urinary VOC Metabolite Analysis

Urinary VOC metabolites were quantified using 2 separate isotope dilution ultra-high-performance liquid chromatography coupled with electrospray ionization tandem mass spectrometry (UPLC-ESI-MS/MS) methods. Metabolites analyzed using the Alwis et al. [30] method were quantitated by diluting 50 µL of urine and 25 µL of internal standard solution to 500 µL with 15 mM ammonium acetate for analysis on a UPLC (I-Class Acquity UPLC system, Waters Inc., Milford, MA, USA) coupled with an ESI-MS/MS (Sciex 5500 triple quad, Sciex, Framingham, MA, USA) operated in negative ion mode. The UPLC was equipped with an Acquity UPLC HHS T3 1.8 um × 2.1 mm × 150 mm (Waters Inc., Milford, MA, USA) column with 15 mM ammonium acetate as solvent A and acetonitrile as solvent B. Lowest reportable values (LRVs), which are the lowest quantifiable values for the assay and are defined as the higher of (1) the lowest calibrator and (2) a precision-based detection limit [31], ranged from 0.5 to 29.5 ng/mL. The calibration curves ranged from 0.157 to 17.1 ng/mL on the low end to 114 to 12,700 ng/mL on the high end, and calibration materials were prepared in 15 mM ammonium acetate in water. Metabolites using the Bhandari et al. [32] method were quantitated by diluting 50 µL of urine and 25 µL of internal standard solution to 500 µL with 5 mM ammonium formate with 0.15% formic acid with pH 2.91 for analysis on a UPLC (I-Class Acquity UPLC system, Waters Inc., Milford, MA, USA) coupled with an ESI-MS/MS (Sciex 5500 triple quad, Sciex, Framingham, MA, USA). The UPLC was equipped with an Acquity UPLC HHS PFP 1.8 µm × 2.1 mm × 100 mm (Waters Inc., Milford, MA, USA) column with a 0.02% formic acid solution as solvent A and methanol as solvent B. LRVs ranged from 0.150 to 64.4 ng/mL. The calibration curves ranged from 0.138 to 30.4 ng/mL on the low end to 119 to 90,800 ng/mL on the high end and calibration materials were prepared in water. For both methods, data was collected using the Analyst software (Sciex, Framingham, MA, USA) and processed using the Multi-Quant software (Sciex, Framingham, MA, USA). Response ratios of analyte to isotopically labeled internal standard were compared to calibration curves that were generated with each batch for quantitation. Calibration materials used were water based due to high endogenous levels of metabolites in urine. Use of non-matrix calibration materials was validated during method development and calibration curve differences between matrix and non-matrix curves were shown to be less than 5% different for both methods. Each batch also contained quality control and blank samples. Values for samples that fell below the LRV threshold were replaced with the midpoint value between 0 and the LRV for each metabolite.

### 2.6. Statistical Analyses

Statistical analyses were conducted and figures were constructed in R Version 2.2.2 [33]. Cannabinoid concentrations in milk, mean milk ∆^9^-THC concentrations after initial cannabis use, milk ∆^9^-THC area under the curve (AUC) after initial cannabis use and estimated average infant daily Δ^9^-THC intake via breastfeeding were calculated previously [10]. Spearman’s rank correlations were conducted to assess associations between cannabinoid concentrations in milk and VOC metabolite concentrations in infant urine. Categorical maternal cannabis use variables were constructed based on reported typical frequency of cannabis use (≥daily vs. <daily) and reported study day cannabis use modality (any smoking vs. only vaping). Given that one of our primary objectives was to assess whether infants exposed to cannabis smoke had higher urinary VOC metabolite concentrations, if a mother recorded any cannabis smoking during the study day, they were classified in the smoker category. Generalized estimating equation (GEE) models were created using the R package *geepack* [34] to determine associations among VOC metabolite concentrations in infant urine at maternal pre- and post-cannabis use, maternal cannabis use modality on the study day (any smoking vs. only vaping), and maternal typical cannabis use frequency (≥daily vs. <daily) while controlling for repeated measures within each mother–infant dyad. Significance was declared at *p* < 0.05.

## 3. Results

### 3.1. Sample and Participant Characteristics

**Figure 2:**
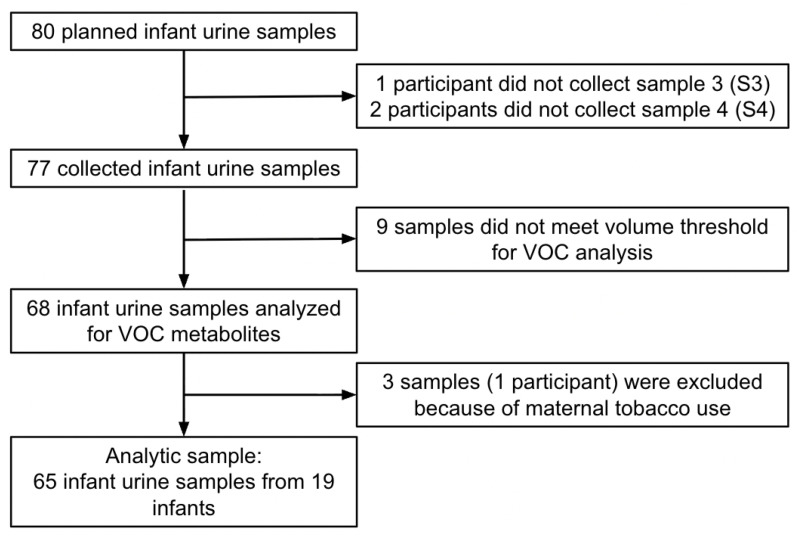
Flowchart of infant urine samples collected and analyzed. The resultant analytic sample was n = 65.

From the originally planned 80 urine samples, we collected 77 samples. Not all participants were able to collect all four urine samples in the allotted time frame; for example, one participant collected samples 15 h and 21 h after cannabis use, far exceeding our study window. Given the variation in sample collection times, we analyzed the concentrations as pre- and post-cannabis use. Of the 77 infant urine samples collected, nine did not meet the volume threshold for analysis (>250 µL). Additionally, one dyad was removed prior to analysis because the mother smoked cigarettes. This resulted in an analytic sample of 65 infant urine samples from 19 mother–infant dyads (Figure 2). One hundred and eleven milk samples collected from their mothers were used to assess maternal cannabinoid concentrations. Holdsworth et al. [10] provide detailed data on milk sample inclusion and exclusion, as well as a detailed write up of the method employed to generate the cannabinoid data. A subset of those data (specifically the Δ^9^-THC concentrations) was used in this study to evaluate potential correlations/associations/relationships of that important cannabinoid with the VOC compounds of particular interest in this investigation.

Mean maternal age and time postpartum at sample collection were 26.9 (±3.2) years and 90.4 (±45.5) days, respectively (Table 1). Sixty-three percent of infants were female (n = 12), twenty-six percent were male (n = 5), and eleven percent were not reported (n = 2). Sixty-three percent of the mothers reported using cannabis on average at least once daily. On the study day, 12 of the 20 mothers used cannabis more than once. Seventeen mothers smoked cannabis at least once, two mothers only vaped, and six mothers both smoked and vaped during the study day. Of the two mothers that only vaped, one reported only ever vaping historically, and one reported that their primary use modality was vaping, although they occasionally smoked at least monthly since giving birth. Each infant’s estimated average daily Δ^9^-THC intake via breastfeeding ranged from 0.002 to 0.196 mg/day (mean = 0.053 mg/day).

### 3.2. Infant Urinary VOC Metabolites Detection

Isotope dilution UPLC-ESI-MS/MS indicated detectable levels (≥LRV) of 24 out of 32 VOC metabolites in infant urine samples (Table 2). Non-baseline urine had greater concentrations in 15 VOC metabolites than their baseline sample (*p* < 0.05). Ten select VOC metabolites (highlighted in Table 2) known to be associated with cigarette smoking behaviors were further analyzed for associations between milk cannabinoid concentrations and cannabis use behaviors and detailed below.

### 3.3. Selected Infant Urinary VOC Metabolite Concentrations and Associations with Maternal and Infant Characteristics and Maternal Cannabis Use Modality and Frequency

MCaMA, 3MHA + 4MHA, and MADA were positively associated with infant age, and MCaMA was positively associated with infant sex. No additional associations were identified between infant age, infant sex, or maternal age and select VOC metabolites (SI Figure S1).

**Figure 3:**
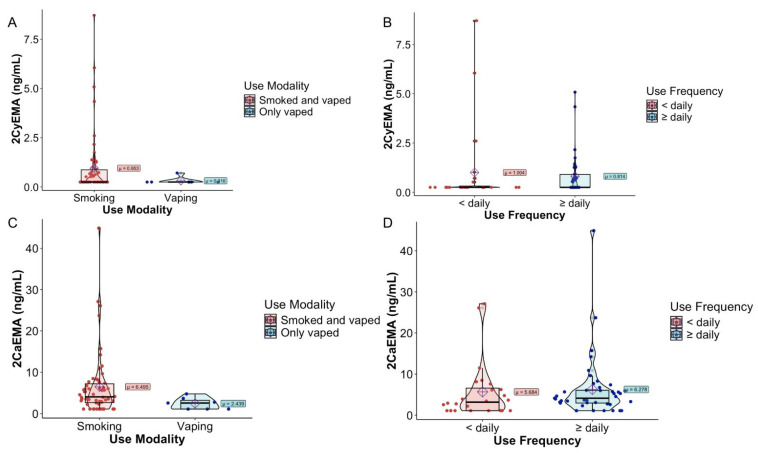
2CyEMA and 2CaEMA concentrations in infant urine by maternal cannabis use. (**A**) 2CyEMA concentration variation by maternal use modality: smoking (n = 58) or vaping (n = 7); (**B**) 2CyEMA concentration variation by maternal frequency of cannabis use: <daily (n = 24) or >daily (n = 41); (**C**) 2CaEMA concentration variation by maternal use modality; (**D**) 2CaEMA concentration variation by maternal frequency of cannabis use. Horizontal lines indicate the median; box hinges indicate 25th and 75th percentiles; whiskers indicate 1.5× interquartile range; points are individual infant data points. We defined “smoking" to include any women who reported smoking, including those who smoked and vaped.

After controlling for repeated measures for each infant, infants of mothers who smoked cannabis on the study day had 89% higher mean 2CyEMA concentrations than infants whose mothers vaped cannabis (B = −0.637, SE = 0.317, *p* = 0.044; Table 3; Figure 3). 2CaEMA concentrations were also greater in the urine produced by infants whose mothers reported any cannabis smoking versus only vaping cannabis on the study day (B = −4.06, SE = 1.42, *p* = 0.004; Table 3). There were no other associations between the selected VOC metabolites and cannabis use modality or frequency characteristics. Analysis of non-selected VOC metabolite associations with maternal cannabis use modality and frequency can be found in SI Figure S2.

### 3.4. Associations Among Selected Infant Urinary VOC Metabolite Concentrations, Milk ∆^9^-THC Concentrations at Baseline, Milk ∆^9^-THC Concentrations After Use, and Milk ∆^9^-THC Area Under the Curve

**Figure 4:**
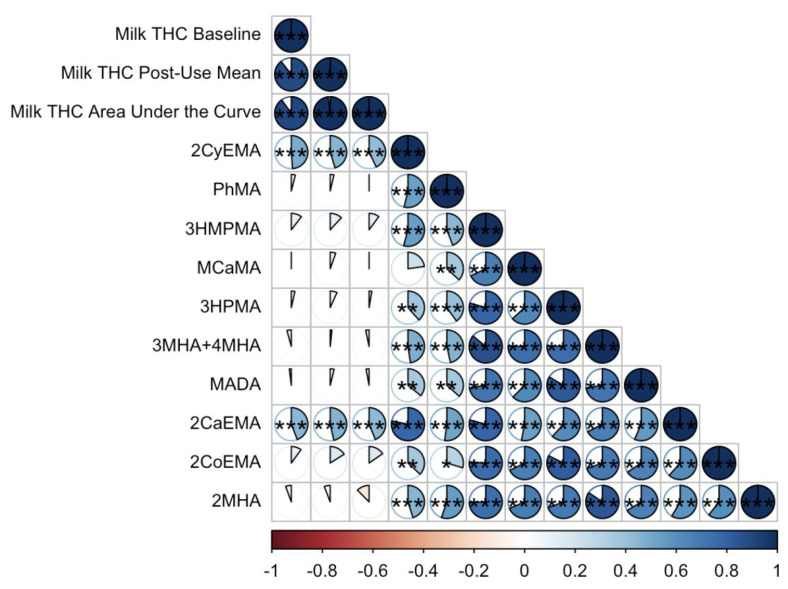
Correlation matrix depicting Spearman correlation coefficients between select VOC metabolites and milk A-THC concentrations (n = 19). Pie charts and color scales, including the solid colors within the circles, visually represent the correlation coefficient (Spearman’s rho). The darker the blue, the stronger the positive correlation; the darker the red, the stronger the negative correlation. * *p* < 0.05, ** *p* < 0.01, *** *p* < 0.001.

2CyEMA and 2CaEMA concentrations in infant urine were higher after maternal cannabis use (B = 0.644, SE = 0.324, *p* = 0.047; B = 3.181, SE = 1.609, *p* = 0.048, respectively; Table 3) than after abstention. 2CyEMA concentrations in urine were positively correlated with ∆^9^-THC concentrations in milk collected at baseline [r(65) = 0.49, *p* < 0.001], mean ∆^9^-THC concentrations in milk collected after use [r(65) = 0.45, *p* < 0.001], and AUC of ∆^9^-THC concentrations of milk after use [r(65) = 0.43, *p* < 0.001, Figure 4]. 2CaEMA concentrations were positively correlated with milk ∆^9^-THC concentrations at baseline (r(65) = 0.44, *p* < 0.001), milk ∆^9^-THC mean post-use concentrations (r(65) = 0.46, *p* < 0.001), and milk ∆^9^-THC area under the curve (r(65) = 0.44, *p* < 0.001, Figure 4). None of the other ten selected VOC metabolites were correlated with ∆^9^-THC concentrations of milk (Figure 4). Associations of non-selected VOC metabolites in urine and ∆^9^-THC concentrations in milk collected at baseline, mean ∆^9^-THC concentration in milk collected after use, and AUC of ∆^9^-THC in milk after use can be found in SI Figure S3.

## 4. Discussion

There were detectable levels of 24 VOC metabolites in the urine of breastfed infants whose mothers used cannabis, including our metabolites of interest. Additionally, all infants had at least one urine sample with detectable VOC metabolite concentrations, indicating exposure to VOC in early life.

To contextualize the VOC metabolite values in our study, we turned to the closest comparison available, the National Exposure Report 2017–2018 [35], for the demographic category of children aged 3–5 years. While it provides an imperfect comparison due to substantial participant age and exposure differences, the report was designed by the National Health and Nutrition Examination Survey (NHANES) to be a nationally representative statistic for biomarker concentrations, with values provided in the report including both smoke-exposed and non-smoke-exposed children. The NHANES report indicated that the calculated U.S. average VOC metabolite geometric mean for children aged 3–5 years was ≥40% higher for the majority of the 32 VOC metabolites we measured than in our current study’s sample of much younger children (infants <6 months of age; Table 2).

Among the selected VOC metabolites that we studied, our results indicated relationships between 2CyEMA and 2CaEMA concentrations in infant urine and maternal cannabis use and Δ^9^-THC concentrations in milk. Infant urinary VOC metabolite concentrations were higher following mothers’ initial cannabis use compared to baseline levels measured after at least 12 h of maternal cannabis abstention. While not indicating causation, it is possible that VOC exposure occurs through breastfeeding and could be dose-dependent, though further research would be needed to determine dose-dependence. Supporting this hypothesis is our finding that infants of mothers with higher ∆^9^-THC concentrations in their milk, at baseline and after cannabis use, had greater concentrations of 2CyEMA and 2CaEMA in their urine.

Infants of mothers who smoked cannabis, rather than solely vaped cannabis, exhibited higher urinary 2CyEMA and 2CaEMA concentrations. This suggests that breastfed infants whose mothers smoke cannabis may be exposed to greater concentrations of VOCs such as known carcinogenic acrylamide, the parent compound of 2CaEMA, and carcinogenic acrylonitrile, the parent VOC of 2CyEMA. Ashley et al. [16] also compared circulating 2CyEMA levels in adults (18–50 years of age) after smoking, vaping, or oral ingestion of cannabis, and found increased concentrations of 2CyEMA (acrylonitrile) after smoking versus vaping or consuming cannabis. Vaping heats cannabis at a lower temperature than combustion, thus acrylonitrile and acrylamide may not be generated or volatilized and released into the inhaled aerosol at such a heat threshold while vaping. Thus, while our data suggest that vaping likely exposes infants to acrylonitrile (2CyEMA) and acrylamide (2CaEMA), this exposure may be greater when breastfeeding women smoke cannabis, as opposed to vaping cannabis.

Although there are no published corollaries between product (e.g., tobacco, cannabis) or age group (e.g., adult or infant) to match this study, there are studies that showcase how smoking increases exposure risk to certain VOCs. For example, St. Helen et al. [36] reported that VOC metabolite concentrations in urine were higher during smoking compared with vaping in 36 dual users (women) of e-cigarettes and cigarettes after two days of only vaping or smoking. In another study examining concurrent tobacco and cannabis use, Smith et al. [37] reported higher concentrations of acrylonitrile in groups using both tobacco and cannabis compared to those that did not use cannabis. Those that used both tobacco and cannabis also exhibited significantly higher levels of acrylamide compared to tobacco-only users. However, it is important to not solely rely on comparison studies, as there remains a lack of data on the effect of VOC exposure on infants.

It is generally accepted that there are negative health implications to acrylonitrile and acrylamide exposure. Acrylonitrile is a carcinogen, with common exposure sources ranging from tobacco smoke to industrial sources and hazardous waste sites. It is widely used in the manufacturing of plastics, acrylic fibers, and synthetic rubber. Common acrylamide exposure comes from tobacco smoke, in addition to contaminated well water, and contains glycidamide, which relates directly to acrylamide’s carcinogenicity [38,39]. While these compounds are associated with cancer in adults, the health risks of exposure via breastfeeding and/or human milk, second, and third-hand smoke, as well as the health risks of these measured doses during early development, are unknown.

Limitations of this study include that the original study aims were not focused on VOC exposure. As such, we did not collect data and/or samples in a manner that could exclude or limit exogenous VOC exposure. For instance, unaccounted-for household smoke exposure, diet, and other household sources may be a potential source of infant urinary VOC metabolites. Furthermore, the original study was not statistically powered a priori to test differences across smoking vs. vaping groups. We, therefore, had far fewer participants who only vaped versus smoked, which could make the distribution of VOC metabolite concentrations in this group appear smaller. Furthermore, as mothers used their own cannabis products, dose standardization was not possible in this study. While the current study cannot assess whether exposure occurred via human milk and/or other exposures, associations between infant urinary VOC metabolites and Δ^9^-THC concentrations in their mothers’ milk suggest that human milk may be an exposure pathway. Future research would also benefit from larger sample sizes to compare differences between subgroups and the addition of urine samples collected from a matched group of infants whose mothers do not use cannabis. Additionally, the variability in the detection half-life of VOC metabolites within individuals is important to consider, as the detection half-life of different VOC metabolites varies, and time from maternal cannabis use to breastfeeding and infant metabolism will all factor into accurate exposure assessment [16]. This study is a first step in a much-needed line of inquiry. Importantly, no conclusions should be drawn regarding the safety or risk of maternal cannabis use during lactation on infant health and wellbeing.

## 5. Conclusions

In summary, this study demonstrates, for the first time, that a wide array of VOC metabolites can be detected and quantified in urine produced by breastfed infants whose mothers use cannabis. Further research is needed to better evaluate whether human milk transfers VOC to the infant or if the exposure is by another route. In addition, potential health risks (or lack, thereof) of second- and thirdhand cannabis exposure on infants and its potential long-term health outcomes must be investigated.

## Figures and Tables

**Table 1:** Selected maternal–infant dyad characteristics ^a^.

Variable	Measure	Value
Infant age (days)	Mean (SD)	90.4 (45.5)
	Median	98
	Range	19–180
Infant sex (n, %)	Male	5 (26%)
	Female	12 (63%)
	Unreported	2 (11%)
Maternal age (years)	Mean (SD)	26.9 (3.2)
	Median	27
	Range	22–37
Milk ∆^9^-THC at baseline (ng/mL)	Mean (SD)	32.30 (40.29)
	Median	21.79
	Range	0.90–137.47
Milk ∆^9^-THC area under the curve/hour	Mean (SD)	6790.16 (7832.66)
	Median	2653.13
	Range	153.63–22,198.59
Maternal mode of cannabis use on study day	Smoking	11 (58%)
	Vaping	2 (11%)
	Both	6 (31%)
Maternal frequency of cannabis use	<Daily	7 (37%)
	≥Daily	12 (63%)

^a^ n = 19 dyads.

**Table 2:** Volatile organic compound metabolites in urine (ng/mL) of breastfed infants ^a,b^.

Analyte	Geometric Mean	Mean (SD)	Minimum	Median	Maximum	Samples with Detectable Analyte (n)	Quantitation Method Ref #
2MHA	5.31	8.21 (9.59)	<5.00	5.01	47.70	33	30
3MHA + 4MHA	26.92	45.90 (62.74)	<8.00	27.0	367.0	58	30
2CaEMA	3.88	6.06 (7.36)	<2.20	3.71	44.90	50	30
MCaMA	13.70	22.40 (36.75)	<6.26	13.60	274.00	56	30
2CoEMA	16.42	22.66 (19.37)	<6.96	16.20	110.00	57	30
2CyEMA	<0.50	0.88 (1.48)	<0.50	<0.50	8.71	26	30
3HPMA	39.00	53.45 (41.62)	<13.00	39.80	198.00	60	30
3HMPMA	65.90	94.29 (91.30)	11.90	65.00	521.00	65	30
MADA	20.19	27.72 (21.99)	<12.00	23.70	103.00	48	30
PhMA	<0.15	<0.15	<0.15	<0.15	0.27	11	32
12CVMA	<12.60	<12.60	<12.60	<12.60	<12.60	0	30
22CVMA	<13.90	<13.90	<13.90	<13.90	<13.90	0	30
2ATCA	152.16	237.87 (280.57)	<29.50	152.00	1800.0	63	30
BzMA	1.20	2.65 (5.70)	<0.50	1.31	44.20	50	30
1PMA	<1.20	<1.20	<1.20	<1.20	<1.20	0	30
1CyHEMA	<2.60	<2.60	<2.60	<2.60	<2.60	0	30
34HBMA	65.64	83.25 (63.27)	13.20	60.40	303.00	65	30
2CaHEMA	<9.40	<9.40	<9.40	<9.40	11.00	1	30
2HEMA	<0.79	<0.79	<0.79	<0.79	2.70	4	30
2HPMA	<5.30	7.20 (6.77)	<5.30	5.57	31.70	34	30
4HMBeMA	1.33	2.65 (3.76)	<1.20	<1.20	16.10	28	30
4HBeMA	<0.60	0.76 (1.00)	<0.60	<0.60	7.49	27	30
MMA	<5.11	7.12 (9.27)	<5.11	<5.11	67.70	28	30
2HPhEMA	<1.00	<1.00	<1.00	<1.00	<1.00	0	30
PhGA	8.50 < 12.00	12.97 (18.10)	<12.00	<12.00	94.60	13	30
PPhMA	<1.91	<1.91	<1.91	<1.91	<1.91	0	30
122CVMA	<3.00	<3.00	<3.00	<3.00	<3.00	0	30
TTCA	<11.20	<11.20	<11.20	<11.20	<11.20	0	30
HMFA	<36.10	183.35 (1024.71)	<36.10	<36.10	8260.00	11	32
HMFG	<16.0	25.13 (113.76)	<16.00	<16.00	924.00	7	32
MUCA	<9.81	<9.81	<9.81	<9.81	39.30	22	32
N2FG	91.68	388.81 (1976.43)	<64.40	83.10	16,000.00	40	32

^a^ n = 65. ^b^ Shaded VOC metabolites represent selected metabolites associated previously with smoking tobacco [16] and are the focus of our analysis on VOC metabolite associations with maternal cannabinoid variables. Study sample values below the LRV were imputed using the midpoint value between 0 and the LRV for each metabolite. In cases where the mean was below the LRV, the SD was not displayed.

**Table 3:** Generalized estimating equation (GEE) analysis for the effect of cannabis use modality (smoking vs. vaping) and pre-/post-cannabis use on 2CyEMA and 2CaEMA concentrations in infant urine ^a^.

Outcome Variable	Predictor	Estimate (B)	Standard Error (SE)	Wald χ^2^	*p*
2CyEMA (ng/mL)	Intercept	0.953	0.314	9.19	0.002 **
	Cannabis Use Modality, Vaping	−0.637	0.317	4.05	0.044 *
	Intercept	0.408	0.095	18.57	1.6 × 10^−5^ ***
	Pre-/Post-Cannabis Use	0.644	0.324	3.96	0.047 *
2CaEMA (ng/mL)	Intercept	6.50	1.42	20.89	4.9 × 10^−6^ ***
	Cannabis Use Modality, Vaping	−4.06	1.42	8.15	0.004 **
	Intercept	3.71	0.715	26.90	2.1 × 10^−7^ ***
	Pre-/Post-Cannabis Use	3.181	1.609	3.91	0.048 *

^a^ n = 65, * *p* < 0.05, ** *p* < 0.01, *** *p* < 0.001.

## Data Availability

The data presented in this study are available on request from the corresponding author due to participant confidentiality guidelines.

## Supplementary Materials

The following supporting information can be downloaded at https://www.mdpi.com/article/10.3390/ijerph23070905/s1: Figure S1. Correlation matrix depicting Spearman correlation coefficients between select VOC metabolites and maternal and infant characteristics; Figure S2. Correlation matrix depicting Spearman correlation coefficients between non-select VOC metabolites and maternal cannabis use modality and frequency; Figure S3. Correlation matrix depicting Spearman correlation coefficients between non-select VOC metabolites and maternal milk 1–THC concentrations.

## Abbreviations

∆^9^-THC	delta-9-tetrahydrocannabinol
1DCV	N-Acetyl-S-(1,2-dichlorovinyl)-L-cysteine
1PMA	N-Acetyl-S-(n-propyl)-L-cysteine
2ATCA	2-Aminothiazoline-4-carboxylic acid
2CaEMA	N-Acetyl-S-(2-carbamoylethyl)-L-cysteine
2CoEMA	N-Acetyl-S-(2-carboxyethyl)-L-cysteine
2CyEMA	N-Acetyl-S-(2-cyanoethyl)-L-cysteine
2DCV	N-Acetyl-S-(2,2-dichlorovinyl)-L-cysteine
2MHA	2-Methylhippuric acid
34HBMA	N-Acetyl-S-(3,4-dihydroxybutyl)-L-cysteine
3HMPMA	N-Acetyl-S-(3-hydroxypropyl-1-methyl)-L-cysteine
3HPMA	N-Acetyl-S-(3-hydroxypropyl)-L-cysteine
3MHA + 4MHA	3-Methylhippuric acid + 4-Methylhippuric acid
AUC	Area Under the Curve
BMA	N-Acetyl-S-(benzyl)-L-cysteine
CDC	Centers for Disease Control and Prevention
CYHA	N-Acetyl-S-(1-cyano-2-hydroxyethyl)-L-cysteine
DFAQ-CU	Daily Sessions, Frequency, Age of Onset, and Quantity of Cannabis Use Inventory
GAMA	N-Acetyl-S-(2-carbamoyl-2-hydroxyethyl)-L-cysteine
GEE	Generalized estimating equation
HMFA	5-Hydroxymethyl-2-furoic acid
HMFG	5-Hydroxymethyl-2-furoylglycine
HEMA	N-Acetyl-S-(2-hydroxyethyl)-L-cysteine
HPM2	N-Acetyl-S-(2-hydroxypropyl)-L-cysteine
IPM3	N-Acetyl-S-(4-hydroxy-2-methyl-2-buten-1-yl)-L-cysteine
LAC Study	Lactation and Cannabis Study
LC-MS	Liquid Chromatography-Mass Spectrometry
LRV	Lowest Reportable Value
MADA	Mandelic acid
McaMA	N-Acetyl-S-(N-methylcarbamoyl)-L-cysteine
MHB3	N-Acetyl-S-(4-hydroxy-2-buten 1-yl)-L-cysteine
MMA	N-Acetyl-S-methyl-L-cysteine
MUCA	Muconic acid
N2FG	N-2-Furoylglycine
NHANES	National Health and Nutrition Examination Survey
PHGA	Phenylglyoxylic acid
PHMA	N-Acetyl-S-(phenyl)-L-cysteine
PHEM	N-Acetyl-S-(1-phenyl-2-hydroxyethyl)-L-cysteine + N-Acetyl-S-(2-phenyl-2-hydroxyethyl)-L-cysteine
PPMA	N-Acetyl-S-(6-hydroxy-2,4-cyclohexadiene-1-yl)-L-cysteine
TCVM	N-Acetyl-S-(trichlorovinyl)-L-cysteine
TTCA	2-Thioxothiazolidine-4-carboxylic acid
UPLC-ESI-MS/MS	Ultrahigh-performance liquid chromatography electrospray ionization tandem mass spectrometry
VOC	Volatile Organic Compounds

## References

[1] Cameron L.D., Fleszar-Pavlović S.E., Yepez M., Manzo R.D., Brown P.M. (2022). Beliefs about marijuana use during pregnancy and breastfeeding held by residents of a Latino-majority, rural region of California. J. Behav. Med..

[2] Passey M.E., Sanson-Fisher R.W., D’Este C.A., Stirling J.M. (2014). Tobacco, alcohol and cannabis use during pregnancy: Clustering of risks. Drug Alcohol Depend..

[3] Schempf A.H., Strobino D.M. (2008). Illicit drug use and adverse birth outcomes: Is it drugs or context?. J. Urban Health.

[4] Thompson R., DeJong K., Lo J. (2019). Marijuana use in pregnancy: A review. Obstet. Gynecol. Surv..

[5] Smith C.B., Schmidt J., Holdsworth E.A., Caffé B., Brooks O., Williams J.E., Gang D.R., McGuire M.A., McGuire M.K., Barbosa-Leiker C. (2024). Cannabis use, decision making, and perceptions of risk among breastfeeding individuals: The lactation and cannabis (LAC) study. J. Cannabis Res..

[6] Bertrand K.A., Hanan N.J., Honerkamp-Smith G., Best B.M., Chambers C.D. (2018). Marijuana use by breastfeeding mothers and cannabinoid concentrations in breast milk. Pediatrics.

[7] Ryan S.A., Ammerman S.D., O’connor M.E., Gonzalez L., Patrick S.W., Quigley J., Walker L.R., Meek J.Y., Committee on Substance Use and Prevention, Section on Breastfeeding (2018). Marijuana use during pregnancy and breastfeeding: Implications for neonatal and childhood outcomes. Pediatrics.

[8] Seabrook J.A., Biden C.R., Campbell E.E. (2017). Does the risk of exposure to marijuana outweigh the benefits of breastfeeding? A systematic review. Can. J. Midwifery Res. Pract..

[9] Baker T., Datta P., Rewers-Felkins K., Thompson H., Kallem R., Hale T. (2018). Transfer of inhaled cannabis into human breast milk. Obstet. Gynecol..

[10] Holdsworth E.A., Berim A., Gang D.R., Williams J.E., Smith C.B., Caffé B., Brooks O., Barbosa-Leiker C., McGuire M.A., McGuire M.K. (2024). Human milk cannabinoid concentrations and associations with maternal factors: The lactation and cannabis (LAC) study. Breastfeed. Med..

[11] Sempio C., Wymore E., Palmer C., Bunik M., Henthorn T.K., Christians U., Klawitter J. (2021). Detection of cannabinoids by LC-MS-MS and ELISA in breast milk. J. Anal. Toxicol..

[12] Wymore E.M., Palmer C., Wang G.S., Metz T.D., Bourne D.W.A., Sempio C., Bunik M. (2021). Persistence of Δ-9-Tetrahydrocannabinol in human breast milk. JAMA Pediatr..

[13] Josan C., Shiplo S., Fusch G., Raha S., Shea A.K. (2023). Cannabis use during lactation may alter the composition of human breast milk. Pediatr. Res..

[14] Astley S.J., Little R.E. (1990). Maternal marijuana use during lactation and infant development at one year. Neurotoxicol. Teratol..

[15] Tennes K., Avitable N., Blackard C., Boyles C., Hassoun B., Holmes L., Kreye M. (1985). Marijuana: Prenatal and Postnatal Exposure in the Human: (496932006-005).

[16] Ashley D.L., De Jesús V.R., Abulseoud O.A., Huestis M.A., Milan D.F., Blount B.C. (2020). Urinary acrylonitrile metabolite concentrations before and after smoked, vaporized, and oral cannabis in frequent and occasional cannabis users. Int. J. Environ. Res. Public Health.

[17] International Agency for Research on Cancer (2004). Tobacco Smoke and Involuntary Smoking: This Publication Represents the Views and Expert Opinions of an IARC Working Group on the Evaluation of Carcinogenic Risks to Humans, Which Met in Lyon, 11–18 June 2002.

[18] Snyder R. (2012). Leukemia and Benzene. Int. J. Environ. Res. Public Health.

[19] Torres S., Merino C., Paton B., Correig X., Ramírez N. (2018). Biomarkers of exposure to secondhand and thirdhand tobacco smoke: Recent advances and future perspectives. Int. J. Environ. Res. Public Health.

[20] Schauer G.L., King B.A., Bunnell R.E., Promoff G., McAfee T.A. (2016). Toking, vaping, and eating for health or fun: Marijuana use patterns in adults, U.S., 2014. Am. J. Prev. Med..

[21] Maertens R.M., White P.A., Rickert W., Levasseur G., Douglas G.R., Bellier P.V., McNamee J.P., Thuppal V., Walker M., Desjardins S. (2009). The genotoxicity of mainstream and sidestream marijuana and tobacco smoke condensates. Chem. Res. Toxicol..

[22] Meier E., Tessier K.M., Luo X., Dick L., Thomson N.M., Hecht S.S., Carmella S.G., Murphy S., Hatsukami D.K. (2021). Cigarette smokers versus cannabis smokers versus co-users of cigarettes and cannabis: A pilot study examining exposure to toxicants. Nicotine Tob. Res..

[23] Moir D., Rickert W.S., Levasseur G., Larose Y., Maertens R., White P., Desjardins S. (2008). A comparison of mainstream and sidestream marijuana and tobacco cigarette smoke produced under two machine smoking conditions. Chem. Res. Toxicol..

[24] Wei B., Alwis K.U., Li Z., Wang L., Valentin-Blasini L., Sosnoff C.S., Xia Y., Conway K.P., Blount B.C. (2016). Urinary concentrations of PAH and VOC metabolites in marijuana users. Environ. Int..

[25] Kenwood B.M., Zhu W., Zhang L., Bhandari D., Blount B.C. (2022). Cigarette smoking is associated with acrylamide exposure among the U.S. population: NHANES 2011–2016. Environ. Res..

[26] Harris P.A., Taylor R., Thielke R., Payne J., Gonzalez N., Conde J.G. (2009). Research electronic data capture (REDCap)—A metadata-driven methodology and workflow process for providing translational research informatics support. J. Biomed. Inform..

[27] Cuttler C., Spradlin A. (2017). Measuring cannabis consumption: Psychometric properties of the Daily Sessions, Frequency, Age of Onset, and Quantity of Cannabis Use Inventory (DFAQ-CU). PLoS ONE.

[28] Britch S.C., Wiley J.L., Yu Z., Clowers B.H., Craft R.M. (2017). Cannabidiol-Δ9-tetrahydrocannabinol interactions on acute pain and locomotor activity. Drug Alcohol Depend..

[29] Young M.S., Martin J.T. (2010). Optimized SPE for UPLC-MS/MS and GC-MS/MS Determination of THC and Its Metabolites in Urine and Blood (Application Note No. 720003738EN).

[30] Alwis K.U., Blount B.C., Britt A.S., Patel D., Ashley D.L. (2012). Simultaneous analysis of 28 urinary VOC metabolites using ultra high performance liquid chromatography coupled with electrospray ionization tandem mass spectrometry (UPLC-ESI/MSMS). Anal. Chim. Acta.

[31] Taylor J.K. (1987). Quality Assurance of Chemical Measurements.

[32] Bhandari D., McCarthy D., Biren C., Movassaghi C., Blount B.C., De Jesús V.R. (2019). Development of a UPLC-ESI-MS/MS method to measure urinary metabolites of selected VOCs: Benzene, cyanide, furfural, furfuryl alcohol, 5-hydroxymethylfurfural, and N-methyl-2-pyrrolidone. J. Chromatogr. B.

[33] Paradis E., Claude J., Strimmer K. (2004). APE: Analyses of phylogenetics and evolution in R language. Bioinformatics.

[34] Højsgaard S., Halekoh U., Yan J. (2002). geepack: Generalized Estimating Equation Package. https://CRAN.R-project.org/package=geepack.

[35] National Center for Environmental Health (U.S.) National Report on Human Exposure to Environmental Chemicals. https://stacks.cdc.gov/view/cdc/133100.

[36] St Helen G., Liakoni E., Nardone N., Addo N., Jacob P., Benowitz N.L. (2020). Comparison of systemic exposure to toxic and/or carcinogenic volatile organic compounds (VOC) during vaping, smoking, and abstention. Cancer Prev. Res..

[37] Smith D.M., O’connor R.J., Wei B., Travers M., Hyland A., Goniewicz M.L. (2020). Nicotine and toxicant exposure among concurrent users (co-users) of tobacco and cannabis. Nicotine Tob. Res..

[38] National Toxicology Program 15th Report on Carcinogens. https://ntp.niehs.nih.gov/whatwestudy/assessments/cancer/roc.

[39] Boettcher M.I., Angerer J. (2005). Determination of the major mercapturic acids of acrylamide and glycidamide in human urine by LC–ESI-MS/MS. J. Chromatogr. B.

