# Supplementary figures and images for: Volatile Organic Compound Metabolites Are Found in the Urine of Breastfed Infants Whose Mothers Use Cannabis: The Lactation and Cannabis (LAC) Study
Source: Int J Environ Res Public Health. 2026 Jul 15;23(7):905. doi: 10.3390/ijerph23070905 (PMC13410178; doi:10.3390/ijerph23070905)

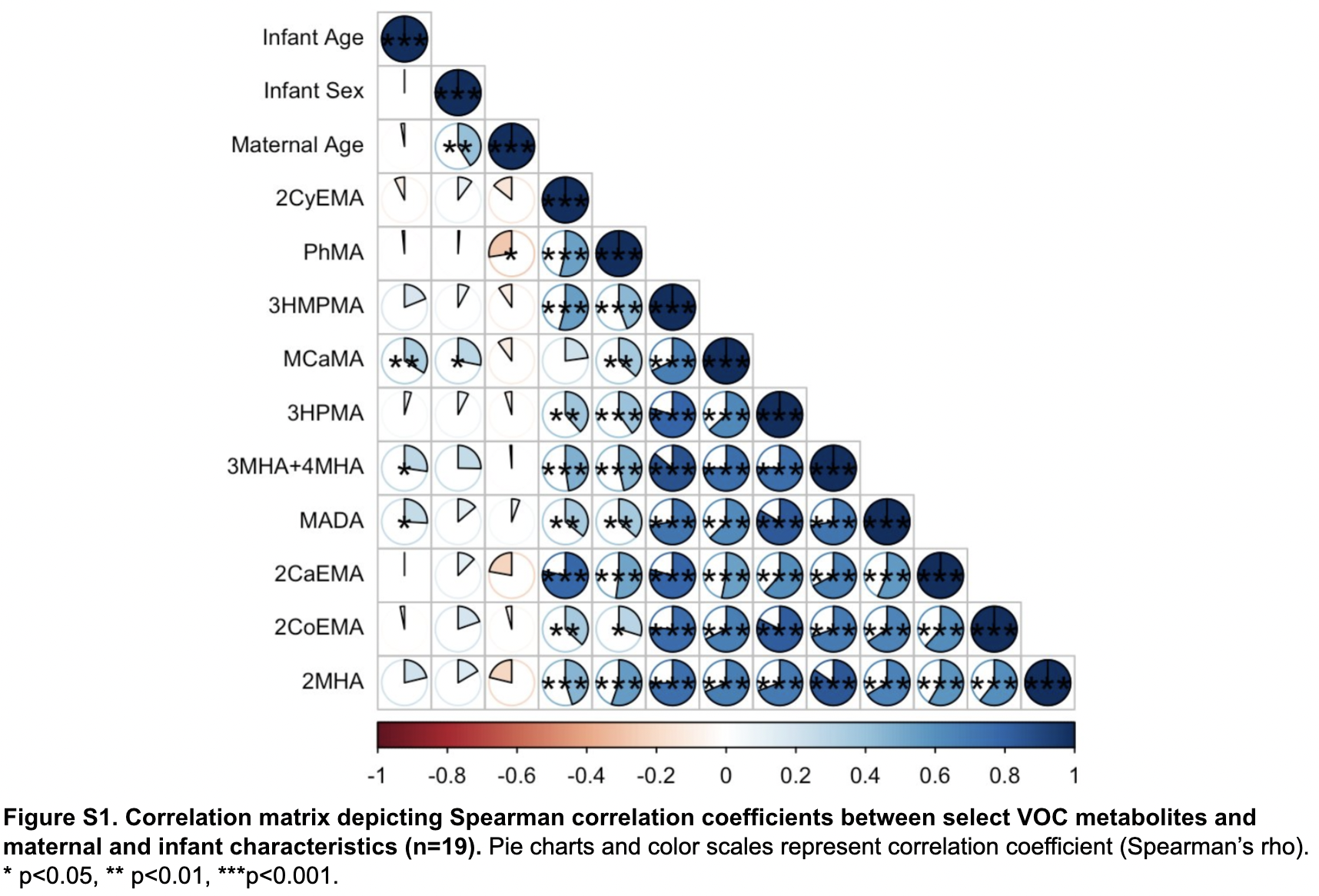

Supplement: Supplementary file 1 [file ijerph-23-00905-s001.zip › Figure S1.png]

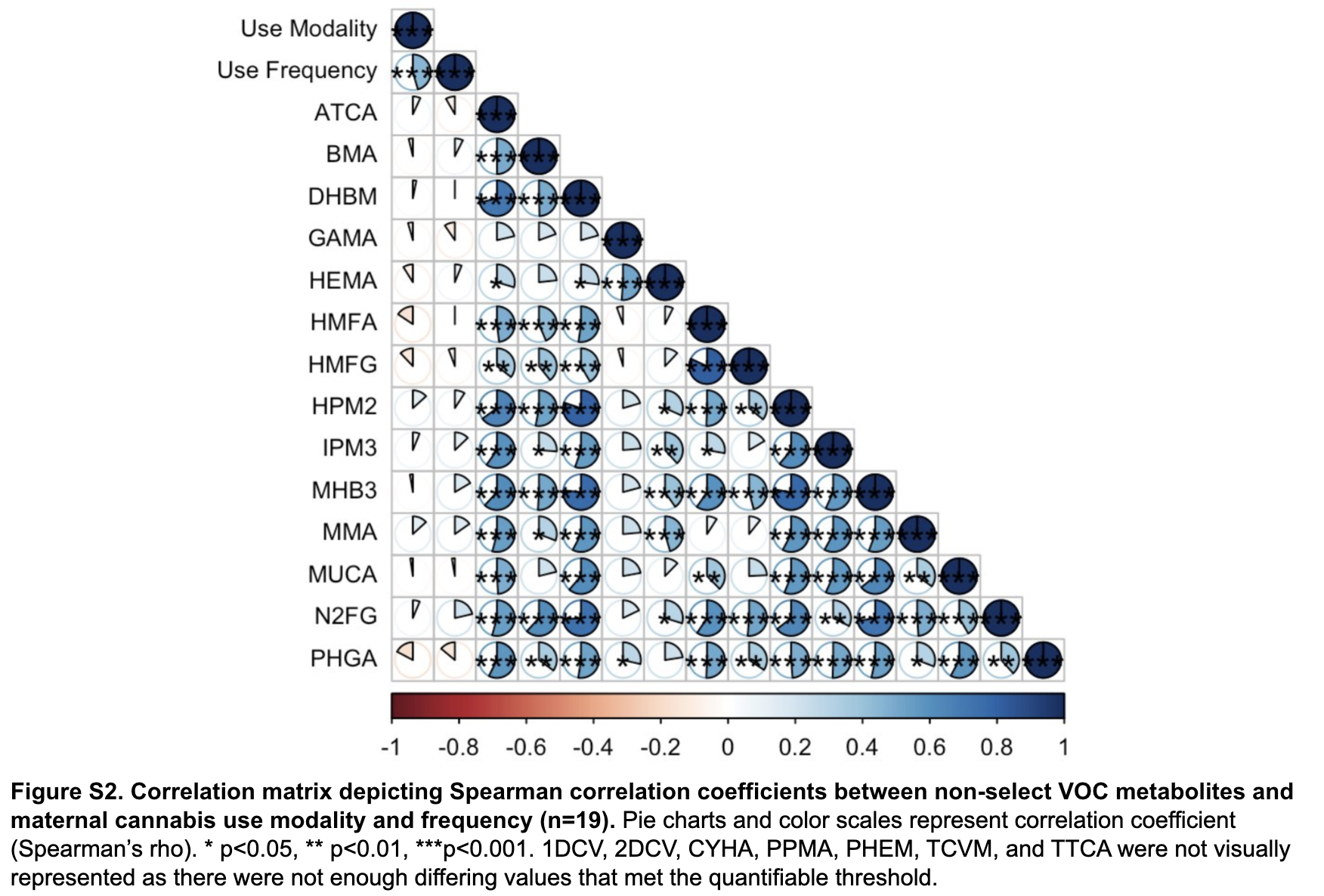

Supplement: Supplementary file 1 [file ijerph-23-00905-s001.zip › Figure S2.png]

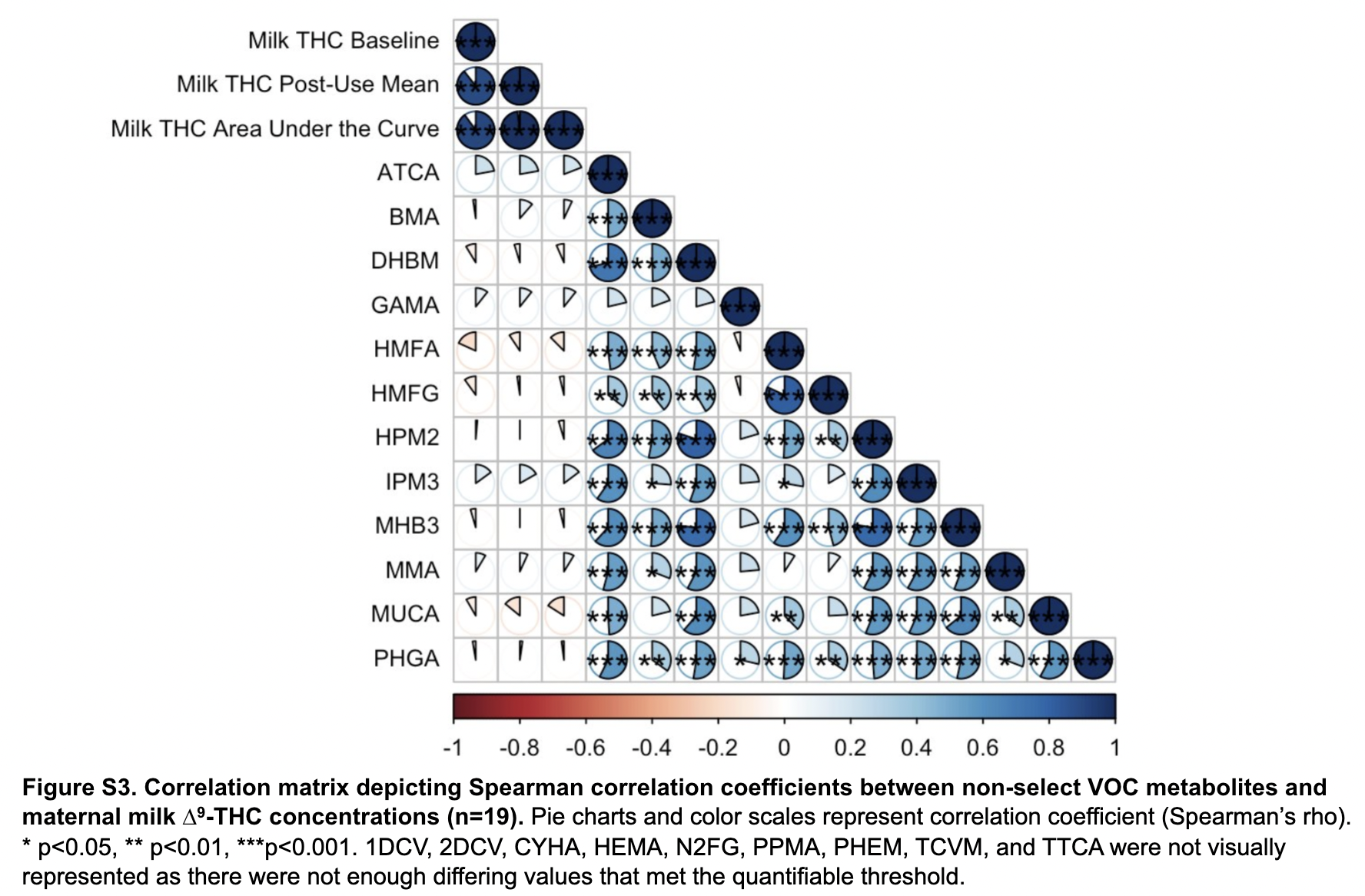

Supplement: Supplementary file 1 [file ijerph-23-00905-s001.zip › Figure S3.png]
